# The first DNA barcode library of Chironomidae from the Tibetan Plateau with an evaluation of the status of the public databases

**DOI:** 10.1002/ece3.9849

**Published:** 2023-02-27

**Authors:** Wu Han, Hongqu Tang, Lili Wei, Enlou Zhang

**Affiliations:** ^1^ State Key Laboratory of Lake Science and Environment, Nanjing Institute of Geography and Limnology Chinese Academy of Science Nanjing China; ^2^ University of Chinese Academy of Sciences Beijing 100039 China; ^3^ Life Science and Technology College Jinan University Guangzhou China

**Keywords:** China, cryptic diversity, DNA barcoding, integrative taxonomy, nonbiting midges, Tibetan Plateau

## Abstract

The main aim of this study was to curate a COI barcode library of Chironomidae from the Tibetan Plateau (TP) as an essential supplement to the public database. Another aim is to evaluate the current status of the public database of Chironomidae in aspects of taxonomic coverage, geographic representation, barcode quality, and efficiency for molecular identification, the Tibetan Plateau, China. In this study, 512 individuals of Chironomidae from the TP were identified based on morphological taxonomy and barcode analysis. The metadata of public records of Chironomidae were downloaded from the BOLD, and the quality of the public barcodes was ranked using the BAGS program. The reliability of the public library for molecular identification was evaluated with the newly curated library using the BLAST method. The newly curated library comprised 159 barcode species of 54 genera, of which 58.4% of species were likely new to science. There were great gaps in the taxonomic coverage and geographic representation in the public database, and only 29.18% of barcodes were identified at the species level. The quality of the public database was of concern, with only 20% of species being determined as concordant between BINs and morphological species. The accuracy of molecular identification using the public database was poor, and about 50% of matched barcodes could be correctly identified at the species level at the identity threshold of 97%. Based on these data, some recommendations are included here for improving barcoding studies on Chironomidae. The species richness of Chironomidae from the TP is much higher than ever recorded. Barcodes from more taxonomic groups and geographic regions are urgently needed to fill the great gap in the current public database of Chironomidae. Users should take caution when public databases are adopted as reference libraries for the taxonomic assignment.

## Graphical abstract

The main aim of this study was to curate a COI barcode library of Chironomidae from Tibetan Plateau (TP) as an essential supplement to the public database. Another aim is to evaluate the current status of the public database of Chironomidae in aspects of taxonomic coverage, geographic representation, barcode quality, and efficiency for molecular identification.

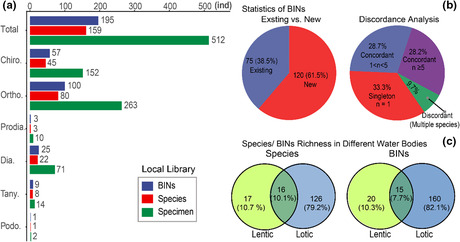

## 1 INTRODUCTION

Understanding regional biodiversity is crucial for effectively conserving and managing biological resources (Ferrier, 2002; Stem et al., 2005). During the past three decades, the inception of the DNA barcoding technique has changed the way of inferring biodiversity from the traditional morphological identification to the effective sequence match method (DeSalle & Goldstein, 2019; Yang et al., 2020). DNA barcoding allows connecting morphological vouchers to a standardized gene fragment and delimitating species according to genetic divergence (Hajibabaei et al., 2007). As the cost‐efficient extension of barcoding, DNA metabarcoding is an emerging approach that could identify multiple species from a mixed sample based on high‐through sequencing of short barcodes (Liu et al., 2020) and has been increasingly applied to biodiversity surveys, biological monitoring, and ecosystem assessment (Compson et al., 2020; Ruppert et al., 2019; Serrana et al., 2019). Compared with traditional morphology, barcoding offers a less subjective approach to identifying organisms, reducing the potential for divergent ecological assessments resulting from individual differences in taxonomic expertise, experience, and opinion of identifiers (Emilson et al., 2017). Though other DNA barcodes have been proposed for molecular identification in various taxa groups, cytochrome c oxidase subunit I (COI) is the most extensively utilized marker gene in animals (Anslan & Tedersoo, 2015). The number of COI barcodes has increased on average by nearly 51% per year since its inception, with a cumulative total of ~2.5 million records in the public database of GenBank (Porter & Hajibabaei, 2018).

The accuracy of barcoding‐based molecular identification is contingent on a comprehensive and high‐quality reference library (Weigand et al., 2019). However, there are still great gaps in taxonomic coverage and genetic diversity in public databases. It is estimated that less than 20% and 5% of the species in plant and animal kingdoms have been represented in the database, respectively (Hebert et al., 2016). If conspecific species are absent in the reference database, the query barcodes will fail to be identified at finer resolution, or worse, return false assignments (Bush et al., 2019; Kvist, 2013). In addition, erroneous identification in the reference sequences is likely to produce wrong taxonomic assignments of query barcodes as well (Paz & Rinkevich, 2021). The Barcode of Life Data system (BOLD, http://www.barcodinglife.org, Ratnasingham & Hebert, 2007) and NCBI GenBank (https://www.ncbi.nlm.nih.gov/, Benson et al., 2017) are two important repositories for DNA barcodes. Due to the periodical exchange between the two libraries, most barcodes are shared by the two databases (Curry et al., 2018). In the current “meta‐biodiversity” era, it is impractical to manually scrutinize the taxonomy of each sequence deposited in the database. Thus, some effective quality filtering processes are embedded in some databases, such as labeling compliant barcode records, flagging probable contamination, and protein‐coding sequences with stop codons on the BOLD (Ratnasingham & Hebert, 2007). Nevertheless, it is inevitable that public databases might accrue considerable erroneous data from various operational and technical faults, such as flawed identification, mislabeling, deficient DNA extraction, and DNA contamination (Lis et al., 2016; Mioduchowska et al., 2018; Paz & Rinkevich, 2021). Moreover, these inaccurate records will likely result in recurrent identification errors, reducing the reliability of related ecological studies (Collins & Cruickshank, 2013).

The chironomid family is one of the most ubiquitous insects with considerable richness and abundance in aquatic ecosystems (Rosenberg, 1992). It is estimated that ~7500 chironomids species of ~550 genera have been accepted in science (Pape et al., 2011), distributed in all geographical regions, including the Antarctic (Rico & Quesada, 2013). Chironomidae are also widely adopted as useful bioindicators in aquatic ecosystems because they are diverse in ecological traits and sensitive to environmental variables (Nicacio & Juen, 2015; Porinchu & MacDonald, 2003). Species‐level identification of aquatic biota has been advocated in freshwater bioassessments because congeneric species can differ substantially in their biological traits (Krosch et al., 2015; Macher et al., 2016). However, chironomids are often identified as coarse taxonomic groups in ecological and paleolimnological studies (Beermann et al., 2018; Van Hardenbroek et al., 2011). This dilemma can be mainly attributed to the great difficulty in the taxonomic work of Chironomidae. Due to their small body size and high diversity, identifying chironomids can be extraordinarily laborious and time‐consuming, even for skilled taxonomists (Jones, 2008). DNA barcoding has also been widely utilized in the identification of chironomids to alleviate the plight of morphological taxonomy. Currently, over 600,000 COI barcodes are deposited on the public database of the BOLD system (as of April 2022). However, the completeness and quality of the existing barcode library of Chironomidae have not been evaluated.

The Tibetan Plateau (TP) is a unique geologic‐geographic‐biotic interactive unit with a surface area of 2.3 million km^2^ and an average elevation exceeding 4500 m (Zhang et al., 2020). As the highest and largest plateau on the planet, the TP is characterized by harsh environments, including extreme coldness, severe aridity, and oxygen deficiency (Wang et al., 2015). It is also a vital speciation center providing diverse habitats with complex topography, heterogeneous climate types, and dramatic physicochemical gradients (Favre et al., 2015). Nowadays, the TP is undergoing the warmest period during the past 2000 years, with air temperature rising at a rate of twice as fast as the global average (Zhang et al., 2020). Evidence from a growing number of studies shows that the TP is experiencing prominent climate change effects, including glacier retreat (Yao et al., 2019), permafrost degradation (Wang et al., 2019), lake expansion (Zhang et al., 2019), and vegetation alteration (Xiong et al., 2019), threatening the habitats of local biota (Liu et al., 2021; Zhang et al., 2021). Chironomids are often the dominant macroinvertebrates of aquatic ecosystems on the TP (Hamerlík et al., 2010; Jiang et al., 2013), but we have little knowledge about species richness because few taxonomic works have been conducted there (Han et al., 2021; Laug et al., 2019; Lin, Chang, et al., 2021; Lin, Mo, et al., 2021; Makarchenko et al., 2022).

In the light of the preceding discussion and the issues raised therein, we aim to (1) investigate the species richness of Chironomidae on the TP and develop a COI barcode library as an important supplement for the public databases; (2) assess the completeness and quality of the public COI barcodes library on the BOLD system; (3) test the efficiency of existing public databases for molecular identification using our new generated barcodes as query sequences; and (4) make some recommendations based on our results that will enhance future barcoding of Chironomidae.

## 2 MATERIALS AND METHODS

### 2.1 Sample collection

**FIGURE 1:**
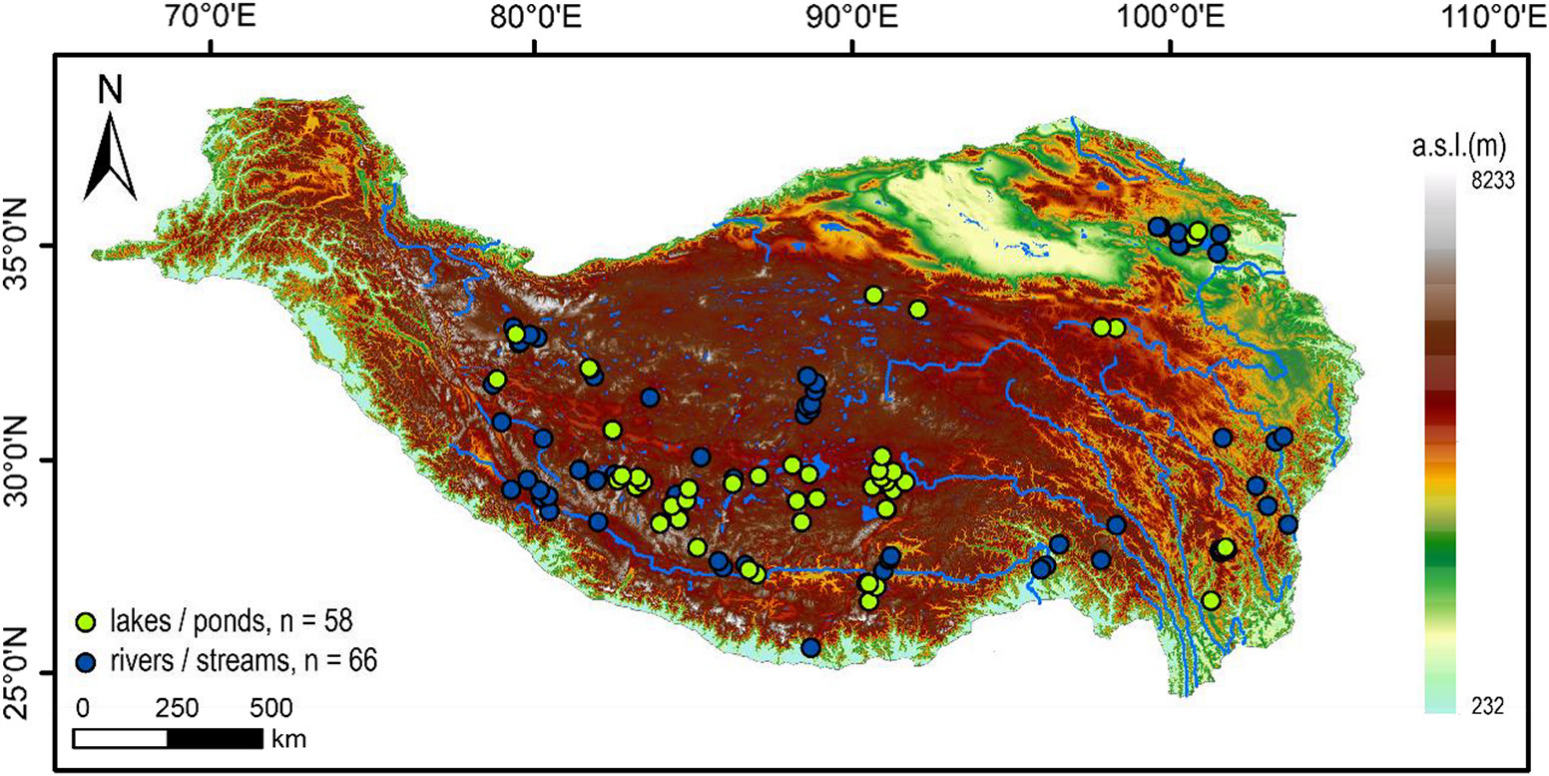
Map showing the location of sampling sites on the Tibetan Plateau, including 58 lentic water bodies (green circles) and 66 lotic water bodies (blue circles).

Specimens were collected from 58 lentic ecosystems (lakes and ponds) and 66 lotic ecosystems (rivers and streams) during the Second Expedition Program of the Tibetan Plateau (STEP) from 2019 to 2021 (Figure 1). Different sampling strategies were adopted for lotic and lentic ecosystems. For lakes and ponds, pupal and larval exuviae and drowned adults were collected with a drift net (mesh size 250 μm) tied to a boat. Living larvae in sediment were collected using a Peterson grab in the open area and a benthic trawl in the nearshore area. For rivers and streams, pupal and larval exuviae were sampled by intercepting running water with dip nets (mesh size 250 μm). Adults were caught using sweep nets along the lake and river banks. All immature materials were washed and filtered in situ, then stored in plastic sealed bags with 95% ethanol, while adults were preserved in 5 mL centrifuge tubes with 85% ethanol.

### 2.2 Molecular experiment

After dissection, the body of larvae and thorax of pupae and adults were transferred to a sterilized centrifuge tube for the molecular experiment. DNA was extracted using the MAGEN® Tissue DNA kit, following the standard protocol provided by the manufacturer. Two universal primers, LCO1490/HCO2198 (Folmer et al., 1994) and C_LepFolF/‐C_LepFolR (Hebert et al., 2004), were adopted to amplify the standard barcode region of COI. Processes and programs for polymerase chain reaction (PCR) were followed as in previous studies (Han & Tang, 2019). Amplification products were verified by agarose gel electrophoresis, then shipped to Sangon Biotech Company, for purification and bidirectional sequencing. Raw sequences were processed following the steps provided by Han and Tang (2019).

All newly generated barcodes and corresponding specimen data and trace files were submitted to the BOLD system and can be seen online through the publicly accessible dataset (DS‐TPCHIR). Voucher slides are deposited in the College of Life Science and Technology, Jinan University.

### 2.3 Taxonomy

An integrated taxonomy strategy was applied for the species identification of collected chironomids. Specimens were firstly dissected and then mounted on microscopic slides using Euparal. Morphological identification was implemented under an optical microscope according to appropriate key tools (Andersen et al., 2013; Langton & Pinder, 2007; Langton & Visser, 2003; Wiederholm, 1983). Some dubious specimens of adult females and immature materials that could not be identified as any known species were labeled with genus name plus coded species name (e.g., *Cricotopus* sp. TP1). The COI barcodes of these specimens were queried against the public database on BOLD and GenBank for molecular identification. However, only matched sequences with more than 98% similarity were treated as reliable results and reconfirmed with morphological knowledge. The Taxon ID Tree tool on BOLD was implemented to construct a neighbor‐joining (NJ) tree of COI sequences using the Kimura 2 Parameter (K2P) model (Kimura, 1980). Specimens with discordant taxonomical assignments and unreasonable phylogenetic positions (i.e., paraphyly, polyphyly, and long branch) in the NJ tree were rechecked under a microscope to exclude potential contamination, misidentification, and mislabels. These processes were repeated until no conflicts between morphological and molecular taxonomy could be detected. This strategy finally ensured that all identified species were highly similar in morphology and monophyletic in phylogeny; however, it is noteworthy that flawed identification, such as cryptic species, may still be present in the curated library.

### 2.4 Barcodes analysis

The optimal threshold (OT) and barcoding efficiency (BE) were determined using *threshold optimization* analysis and *Best Close Match* function of the package *spider* v1.4‐2 in R platform (R Core Team, 2020) according to a standard manual (Brown et al., 2012). The concept of OT was that molecular identification at this threshold produces minimum cumulative errors. BE indicated the percentage of correctly identified sequences at the optimal threshold. More details could be seen in Gadawski et al. (2022).

### 2.5 Assessment of the public COI library on the BOLD system

The specimen data of public barcodes were downloaded from the BOLD system using the *bold_specimens* function of the package *bold* (Chamberlain, 2019). Specimens without COI barcodes were filtered out. Identification and geographic information were compiled from the downloaded checklist to analyze the representation of different taxa and regions. To gauge the congruence status of public barcodes of Chironomidae, the R‐based application, Barcode, Audit & Grade System (BAGS, Fontes et al., 2021), was adopted to qualitatively rank each species in the public database on the BOLD system. BAGS is a qualitative ranking system that assigns one of the five grades (A to E) to each species in the reference library, according to the attributes of the data and congruency of species names with sequences clustered in Barcode Index Numbers (BINs). Fontes et al. (2021) explained the definition of each grade in detail. Briefly, species ranked to Grade A (Consolidated concordance) and Grade B (Basal concordance) means they have at least three barcodes, and all of the barcodes belong to one BIN, but species ranked to Grade A (>10 barcodes) have more barcodes than Grade B (≤10 barcodes). The species in Grade C are assigned to more than one BINs (Multiple BINs). Species with less than 3 barcodes are ranked to Grade D (Insufficient data). Species of grade E share BINs with other species (Discordant species assignment). Only COI barcodes with the species name, BINs, and a minimum length of 500 bp were retained for BAGS analysis.

### 2.6 Test the efficiency of the public database for molecular identification

Haplotypes of the newly curated library were queried against the *nt* database of GenBank, which is commonly used for taxonomic assignment in metabarcoding studies, to test the reliability of barcoding‐based identification for Chironomidae. BLAST searching using the top‐hit strategy (Camacho et al., 2009) was chosen for the taxonomic assignment of query sequences because it is easy‐operated, commonly used, and competes well with more complex approaches (Hleap et al., 2021). There are three possible situations when comparing our taxonomy result with molecular identification. (1) Correct identification, which means the molecular identification result is consistent with our taxonomy result; (2) Wrong identification, which means the molecular identification is conflicted with our taxonomy result at the given taxonomic resolution; (3) Insufficient identification, the top‐hit match was lack of taxonomic information at the given taxonomic level.

## 3 RESULTS

### 3.1 DNA barcodes library of Chironomidae from the TP

**FIGURE 2:**
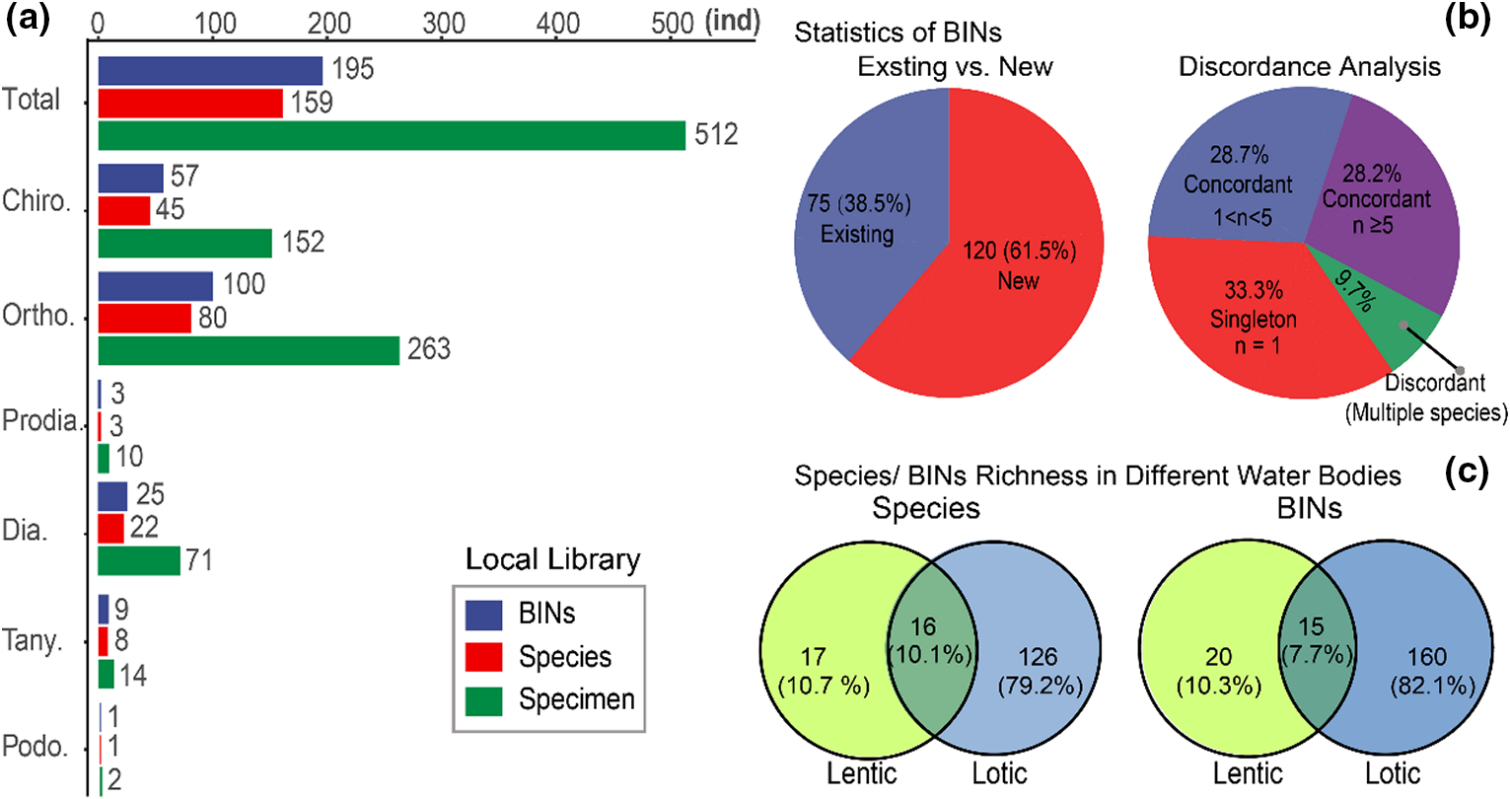
Basic taxa composition information in the newly curated library of Chironomidae from the TP. (a) The number of specimens, species, and BINs in the family and the six subfamilies. Total, Chironomidae; Chiro., Chironominae; Ortho., Orthocladiinae; Prodia., Prodiamesinae; Dia., Diamesa; Tany., Tanypodinae; Podo., Podonominae; (b) the proportion of newly generated and existing BINs (left), and discordance analysis (right), *n* referring to the number of sequences incorporated in the BINs; (c) the proportion and number of species (left) and BINs (right) from lentic and lotic habitats. Concordant BINs, all barcodes are conspecific with more than one barcode; discordant BINs, a BIN contains barcodes of more than one species; singleton BINs, a BIN contains only one sequence.

After stringent taxonomic work, a library comprised of 512 COI barcodes was curated with corresponding geographic information, sequence trace files, and digital photos, representing the first DNA barcode library of Chironomidae from TP. These sequences were free from contamination and stop codons, with an average length of 638 base pairs (range: 500–658 bp). Integrative taxonomy suggested 159 provisional species belonging to 54 genera and six subfamilies (Figure 2a, Table S1), of which 92 barcode species failed to be identified as any known species using either morphological or molecular method, and were temporarily assigned to a coded species name (Table S1). All barcodes were assigned to 192 operational taxonomic units (OTUs) and 195 BINs by RESL and BIN clustering analyses on BOLD, of which 120 (61.5%) BINs were newly formed (Figure 2a,b). The BIN Discordance analysis suggested that 102 (52.3%) BINs were singleton, and the remaining 93 (47.7%) BINs were concordant in the local library. However, 19 (9.7%) BINs were discordant, and singleton BINs decreased to 65 (33.3%) when the public sequences on BOLD were incorporated into the analysis (Figure 2b). Regarding concordant BINs, 55 (28.2%) BINs were represented by at least five sequences (Figure 2b). There were 138 species represented by one BIN, and 21 represented by multiple BINs.

The subfamilies Chironominae, Orthocladiinae, and Diamesinae showed relatively high species richness, with 45, 80, and 22 species, respectively (Figure 2a). By contrast, only a small number of species were observed from the subfamilies Prodiamesinae (*n* = 3), Tanypodinae (*n* = 8), and Podonominae (*n* = 1; Figure 2a). The most common genera were *Cricotopus* (61 barcodes assigned to 12 species), *Chironomus* (56 barcodes assigned to nine species), *Acricotopus* (51 barcodes assigned to 13 species), *Orthocladius* (49 barcodes assigned to 14 species), *Diamesa* (36 barcodes assigned to 13 species), and *Micropsectra* (31 barcodes assigned to 11 species). Species were represented by different numbers of barcodes, with the highest number of specimens for *Chironomus bernensis* (22), *Pseudodiamesa alica* (20), *Cricotopus dentatus* (16), *Orthocladius multidentatus* (15), and *Paracladius akansetus* (15); however, 74 species were represented by a single specimen (Table S1). Interestingly, lotic water bodies (streams or rivers) had much higher species richness than lentic water bodies (lakes or ponds), though they had comparable numbers of sampling sites (Figure 2c). There were 16 (11.3%) species and 15 (7.7%) BINs observed in both types of water bodies (Figure 2c). Adult females of 23 species, pupae of 25, and larvae of 37 were associated with their adult males (Table S1).

### 3.2 Genetic distance and optimal threshold for molecular identification

**FIGURE 3:**
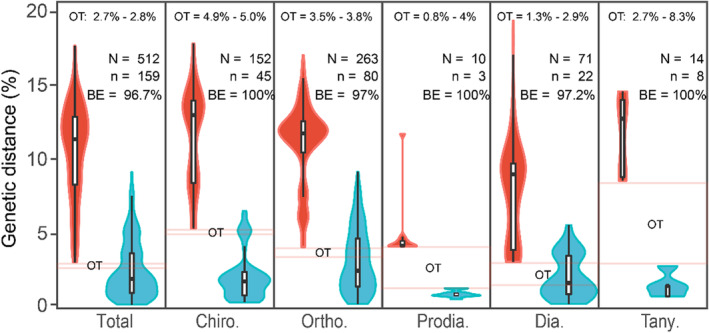
Violin plots of the distribution of inter‐ (red) and intraspecific (blue) genetic distance (K2P) of the 512 barcodes of Chironomidae from the TP. The pink horizontal lines refer to the optimal thresholds for molecular identification. Red, interspecific distance; blue, intraspecific distance; *N*, number of sequences; *n*, number of species; BE, barcoding efficiency.

The maximum intraspecific distance ranged from 1.24% to 7.5%, while the minimum interspecific distance ranged from 3.14% to 8.4% (Table S2). There was no definite “barcode gap” in the family and the subfamilies Chironominae, Orthocladiinae, and Diamesinae, but it could be seen in the poorly represented subfamilies Prodiamesinae and Tanypodinae (Figure 3). In terms of single species, the max and mean intraspecific distances were always lower than the interspecific distances to their nearest neighbors (Figure S1). The optimal threshold (OT) for molecular identification was 2.7%–2.8% in the family with the cumulative errors of 17 sequences and greatly varied among different subfamilies (Table S3). The *Best Close Match* analysis detected 421 correctly identified barcodes, 0 incorrectly identified barcodes, and 91 unmatched barcodes (No ID) at the calculated optimal threshold. After removing singleton species, the efficiency of DNA barcode‐based identification in this library was 96.68%, ranging from 97% to 100% among different subfamilies (Table S3).

### 3.3 Comprehensiveness and quality of the public database

**FIGURE 4:**
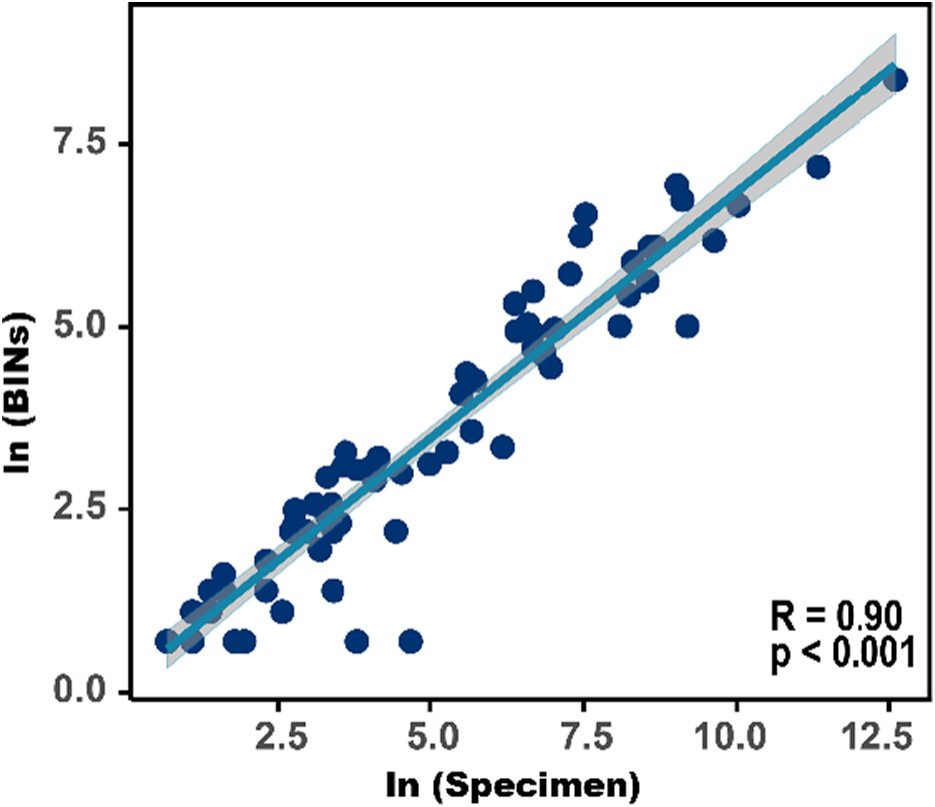
The relationship between the number of specimens and BINs in the public COI database on the BOLD. Notice that the *x*‐axis and *y*‐axis are in scaled for better display.

Over 492,000 COI barcodes of Chironomidae were publicly accessible on the BOLD system, belonging to 222 genera of six subfamilies (Table 1). However, only a small proportion of barcodes were identified at fine taxonomic resolutions, with 52.56% at the genus level and 29.18% at the species level (Table 1). The most commonly encountered three subfamilies, Chironominae, Orthocladiinae, and Tanypodinae, had the highest number of specimens and species richness in the public database on BOLD (Table 1). Comparatively, Diamesinae, Podonominae, Prodiamesinae, and Telmatogetoninae were represented by much fewer specimens (Table 1). The number of recorded species in the database was much lower than that known to science, except that Tanypodinae had more barcode species in the database than accepted species (Table 1). The richness of BINs significantly correlated with the number of specimens (*R* ^2^ = 0.9; *p* < .001; Figure 4).

**TABLE 1:** The summary statistics on the COI barcodes in the public database of Chironomidae on BOLD.

Group	Specimen (*N*)	Genus (*n*)	Genus (%)	Spe. Mol./Acc. (*n*).	Species (%)	BINs (*n*)
Chironominae	94,840	75	88.82	1153/2850	45.12	2765
Orthocladiinae	211,101	76	74.08	808/2468	41.16	2824
Prodiamesinae	167	3	100	19/28	100	20
Diamesinae	2815	13	98.29	92/235	77.48	141
Telmatogetoninae	11	2	100	4/40	100	5
Tanypodinae	21,334	44	67.92	738/610	51.57	656
Podonominae	273	9	99.27	16/160	93.41	27
Chironomidae	492,171	222	52.56	2836/7500	29.18	12,043

*Note*: Genus (%) and species (%) refer to the percentage of barcodes identified to the corresponding taxonomical levels; Spe. Dat./Acc. refers to the number of recorded species on the database (BOLD) and the estimated accepted species.

**FIGURE 5:**
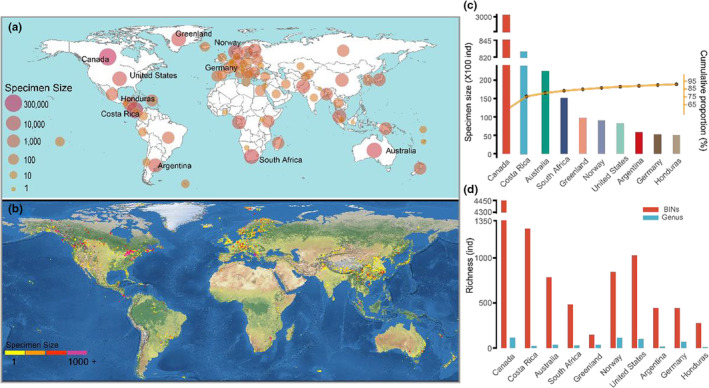
Contribution of each county/region to the COI barcodes of Chironomidae in the public database on BOLD (as of Mar., 2022). (a, b) The map displaying the number of records in each country/region; (c) the specimen size and cumulative proportion of the top 10 countries; (d) the genus and BINs richness of the top 10 countries. Notice that y‐axis was broken for better display.

Barcoding efforts for Chironomidae were extremely uneven among different regions (Table S4, Figure 5). As the birthplace of barcoding, Canada contributed the most barcodes and species richness (BINs) of Chironomidae, accounting for ~60% of the total sequences (Figure 5c,d). The top 10 countries cumulatively contributed to ~94% of the public barcodes on the BOLD (Table S4, Figure 5c). Generally, barcoding studies on Chironomidae were prosperous in North America, Europe, East Asia, and Australia, while relatively poor in South America, Africa, Russia, and Central Asia (Figure 5a,b).

**FIGURE 6:**
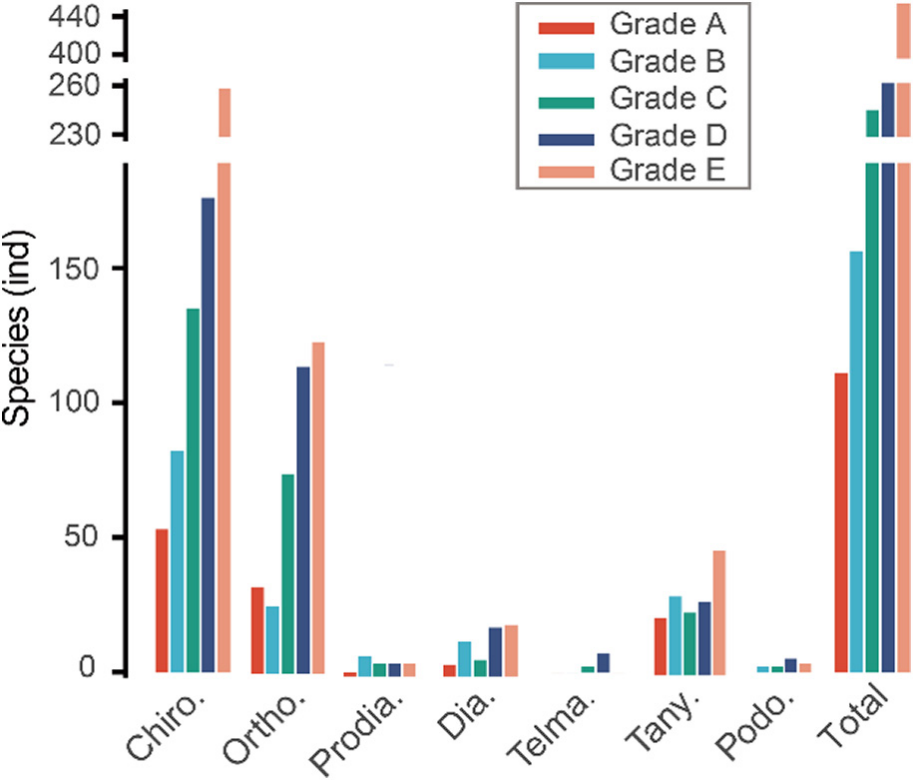
Evaluation of the quality status of the public COI database on BOLD using BAGS. The bar plots displaying the distribution of the number of species assigned to each qualitative grade.

BAGS analysis was conducted to evaluate the quality status of the public COI database of Chironomidae on BOLD. The result showed that the overall quality of the public database was not so optimistic (Table S5, Figure 6). Only a small proportion of species of Chironomidae were ranked to Grade A (8.49%, Consolidated concordant) and Grade B (11.92%, Basic concordant), indicating good congruence between BINs and species. Other species were assigned to Grade C (18.65%, Multiple BINs), Grade D (26.45%, Insufficient data), and Grade E (34.48%, Discordant species assignment). The status of each subfamily was generally similar to the whole family, with most species ranked to Grade D and few species ranked to Grade A and B.

### 3.4 Efficiency of molecular identification using public database

**FIGURE 7:**
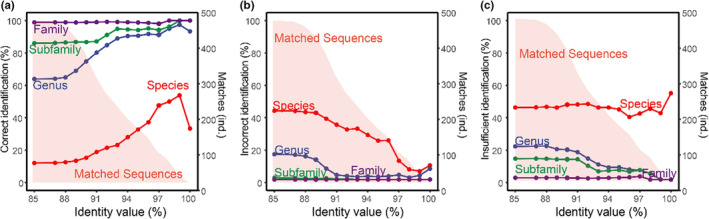
Test on the efficiency of *nt* database for molecular identification using the curated library of Chironomidae from the TP. The dots and lines show the proportion of correct identification (a), incorrect identification (b), and insufficient identification (c) at different taxonomic resolutions; the area chat refers to the proportion of matched sequences. Blast method with a top‐hit strategy was adopted for the taxonomic assignment of query barcodes.

A total of 474 haplotypes from the newly curated COI barcodes were queried against the *nt* database in GenBank for molecular identification. The number of matched sequences sharply decreased with the rise of the identity threshold. All query sequences could be matched at the 85% identity threshold but decreased to 15 sequences at the 100% threshold (Figure 7). The correct identification rate increased at a stricter threshold at the expense of a rapid decrease in the matched sequences and was much higher at coarser taxonomic levels (Figure 7a). About 50%, 91%, and 94% of matched sequences were correctly assigned to species, genus and subfamily levels at the identity value of 97%, which is often adopted in metabarcoding studies. On the other hand, the misidentification rate decreased when the identity threshold was lowered with 22% and 1.8% of matched species wrongly identified at the species and genus levels at the threshold of 95% (Figure 7b). Significantly, over 40% of matched sequences could not be identified to species level at any thresholds because the matched sequences lacked sufficient taxonomic information (Figure 7c).

## 4 DISCUSSION

### 4.1 Species diversity and genetic divergence of Chironomidae from the TP

In this study, a COI barcode library was first curated for Chironomidae on the TP based on integrative taxonomy, by which great species richness was comprehensively recorded. The result suggested that considerable cryptic species have yet to be recognized on the plateau since many specimens could not be assigned to known species. Due to its unique topographical characteristics, the TP is known as a center for speciation and differentiation, accommodating highly endemic diversity (Wu et al., 2022; Yang et al., 2009). However, biodiversity on the TP is far from fully explored, especially for some highly diverse groups (Favre et al., 2015). Recently, more hidden diversity has been revealed with the help of barcoding techniques (Han et al., 2022; Wang et al., 2020; Xu et al., 2018). Furthermore, it has become increasingly urgent to improve our understanding of the species diversity on the Earth's highest plateau for better biological conservation because the unprecedented climate change on the TP has been profoundly altering the natural habitats of local biota (Kou et al., 2020; Xin et al., 2021; Zhang et al., 2021). For example, cold stenothermal members of *Diamesa* in Chironomidae, which inhabit kryon zones of glacier‐fed streams, are under great threat of habitat loss due to the extensive glacier retreat on the TP (Hamerlik & Jacobsen, 2012; Lencioni, 2018).

Although our taxonomic result has been stringently checked, cryptic diversity could not be excluded even in the curated library, as suggested by the huge intraspecific divergence in some barcode species. These species usually comprise specimens collected over a broad spatial scale; thus, the large genetic divergence may reflect the restriction of gene flow caused by geographical isolation (Liu et al., 2019; Wu et al., 2022). However, it is arbitrary to define the species concept by barcode divergence of limited specimens alone without other lines of evidence (Luo et al., 2018; Meier et al., 2021). Here, we conservatively treated these species as conspecific because reliable variations in morphology, ecology, and behavior had not been observed in available materials. Meanwhile, further taxonomic work has been in progress, with the supplement of associated specimens of different life stages. For example, our recent work established two morphologically similar species of *Monodiamesa* based on slight but stable variations in diagnostic features and relatively huge genetic divergence (Han et al., 2021). A similar case could be found in *Diamesa* in another study (Han et al., 2022).

Our observation found much higher species richness in lotic water bodies than in lentic ones at a large spatial scale (Figure 2c). This survey mainly focused on lakes and streams, including a few ponds and rivers. Lakes in the TP are often characterized by relatively high salinity and the absence of hydrophyte, which limits the occurrence of diverse chironomids (Bouchard Jr et al., 2022; Zhang et al., 2007). Comparatively, fresh streams could provide more diverse habitats due to their great divergences in altitude along with gradients in environmental variables, such as glacial influence, groundwater recharge, substrate, water temperature, and oxygen saturation (Hamerlik & Jacobsen, 2012). Insufficient sampling may also reduce the obtained species richness. However, we have made great efforts to survey lake ecosystems, collecting materials of all life stages from littoral zones to open areas. Importantly, lake sediment across the lake depth gradient was also collected to obtain larvae of those species not in the emergence stage. Hence, sampling methods played little role in explaining the low species richness of chironomids in the lentic water bodies. All observed subfamilies showed more species richness in lotic systems, especially for Diamesinae, which had 22 species in lotic ecosystems but two in lentic ecosystems (Table S1). Similar patterns could also be found in the common genera in the TP. Based on our observation, further biodiversity investigation and conservation efforts should pay more attention to the lotic ecosystems.

### 4.2 Barcoding efficiency for molecular identification and OT

The OT value inferred from the dataset of Chironomidae from the TP (OT = 2.7%–2.8%) was higher than the proposed threshold in Alpine chironomids (OT = 0.7%–1.4% Montagna et al., 2016) and European chironomids (OT = 1.6%) but comparable with that in the Lake Skadar region (OT = 2.4%, Gadawski et al., 2022). The relatively high threshold value likely resulted from the poor representation of genetic diversity, which increased the divergence between the query sequence and its nearest neighbor. In fact, a large proportion of species (79.2%) and BINs (62%) had less than five barcodes in the curated library, resulting in 17 barcodes failing to match conspecifics at the inferred optimal threshold value. Nevertheless, barcoding was still confirmed as a reliable tool for molecular taxonomy if conspecific barcodes had been represented in the reference library since no sequence was misidentified at the optimal threshold (Table S3). The inclusion of more specimens will fill the gaps in genetic diversity, thus improving the efficiency of barcoding‐based identification.

### 4.3 Current status of the public library for Chironomidae

It has been widely recognized that obtaining accurate taxonomic identification for the query sequence can be difficult or impossible if comprehensive and high‐quality reference libraries are unavailable (Curry et al., 2018). Here, we evaluated the development status of the current public COI database of Chironomidae on the BOLD. It is evident that great efforts and capital have been spent on barcoding studies of chironomids, as over 600,000 public barcodes had been deposited on the BOLD (as of Apr. 2022). However, the recorded species (*n* = 2836) in the public database only accounted for a small proportion of known chironomids (Table 1), reflecting great gaps in taxonomic coverage in the public barcode database of Chironomidae. The good linear correlation between the number of BINs and specimens also suggested that great hidden species richness of chironomids had not been recorded, even in the best‐sampled regions (Ekrem et al., 2007). Previous studies found that the risk of wrong identification increased when target species were not represented in the reference libraries (Virgilio et al., 2010). When incomplete databases are applied in metabarcoding studies, the rate of false‐positive (FP) and false‐negative (FE) errors will rise in the taxonomic assignment (Ruppert et al., 2019). However, a large amount of cryptic diversity has not been fully explored in the public database since the number of BINs was much higher than the recorded species, partly attributed to the fact that only a small proportion (29.18%) of COI barcodes of Chironomidae had been identified at the species level (Table 1). Comparatively, Porter and Hajibabaei (2018) found that ~43% of freshwater records in the NCBI nucleotide database were fully identified to the species rank. The worse situation in Chironomidae is predictable because identification of this taxa is more difficult and time‐consuming than most aquatic taxa, even for skilled taxonomists (Nicacio & Juen, 2015). However, the frustrating situation may limit the application of Chironomidae in ecological studies because useful biological information will be obscured at coarser taxonomic resolutions (Greffard et al., 2011; Nicacio & Juen, 2015).

The development of barcoding studies of Chironomidae was found to have been extremely unbalanced across different regions (Figure 5). Our observation was in line with previous studies that barcodes of Canadian specimens were disproportionately represented in the databases (Curry et al., 2018; Porter & Hajibabaei, 2018). Generally, barcoding studies were most common in North America and Europe, where significant DNA barcoding campaigns have been conducted (Weigand et al., 2019). The extreme imbalance may lead to an overly optimistic estimation of the development of barcoding of Chironomidae as many poorly studied regions may be obscured. Some biodiversity hotspots, such as the west coast of South America, Indo‐Burma, and Southeast Asia (Myers et al., 2000), which should be the focus of biodiversity research, have contributed very limited COI barcodes of Chironomidae. Given the fact that significant habitat destruction is happening globally, advances in research on Chironomidae of these poorly explored regions would help assess ecological thresholds and the extent of anthropogenic disturbance on aquatic ecosystems (Nicacio & Juen, 2015).

Here, the quality status of the public barcodes of Chironomidae on the BOLD was evaluated using BAGS (Table S4, Figure 6). Our result suggested that the quality of the public database was far from ideal. Only a small proportion of species (20%) was determined as concordant between BINs and morphological species (Grade A and Grade B). Many species (19%) had multiple BINs (Grade C) as a result of large intraspecific distances. Though multiple BINs under one species name may result from slight but consistent variation in barcodes, it may also indicate possible cryptic diversity deserving of further taxonomic verification on these species (Sheffield et al., 2017). About 26% of species were represented by less than three sequences (Grade D). This limited taxonomic sampling may lead to underestimates of intraspecific genetic distance and degrade the efficiency of molecular identification in practice (Luo et al., 2015). It is worth noting that most species (~34%) were ranked to Grade E, meaning multiple species shared a single BIN. Previous studies have shown that DNA barcoding may fail to distinguish some species with distinct morphological and ecological variations (Sheffield et al., 2017). Similar cases could be found in the Chironomidae, such as *Clunio balticus* and *C. ponticus* (Michailova et al., 2021). Lin et al. (2022) noted that the mutation rate of COI was relatively low in the subfamily Diamesinae, thus COI barcodes may fail to define the species boundary of some taxa, such as *Diamesa* (Montagna et al., 2016). However, such cases have not been commonly reported in Chironomidae. Instead, synonyms, mislabels, and misidentifications are more likely to be the main reasons for the chaotic taxonomic information in the public database (Sheffield et al., 2017). Though the concept of BIN is obviously not equal to species (Meier et al., 2021), the incongruence between BINs and species is still a good starting point for revising erroneous data in public libraries (Ratnasingham & Hebert, 2013).

### 4.4 Molecular identification using top‐hit strategy

In this study, the efficiency of the *nt* database for molecular identification was tested using our curated COI reference library (Figure 7). The result suggested that the accuracy and efficiency of molecular identification for Chironomidae were severely limited by the poor quality of the public reference database. The correct identification rate was much lower than in a previous report, in which 53% of query sequences of insect taxa were correctly identified at the species level (Meiklejohn et al., 2019). The poor performance in this study could be partly attributed to the fact that many species in the test library had not been taxonomically described, and their barcodes had not been recorded in the public library, resulting in many query sequences being unable to match conspecific COI barcodes. On the other hand, the prevalence of barcodes with inaccurate or insufficient taxonomic information in the public library also hindered the correct identification of query barcodes at finer resolutions.

In metabarcoding studies, it is crucial to determine taxonomic resolution and identity threshold for molecular identification (Laini et al., 2020). Taxonomy can be assigned using either a fixed and high identity value where only fine resolution assignments (i.e., genus or species) are obtained or a multilevel assignment approach where assignments at multiple taxonomic levels are conducted using different identity value thresholds (Alberdi et al., 2018). Though great bias exists in the curated library (e.g., taxonomic coverage and number of barcodes), our test simulated a common process of molecular identification, and thus inspired us to reasonably take advantage of the public databases. The results showed that taxonomic assignment at coarser resolutions (i.e., genus, subfamily, family) was reliable even at a relatively low identity threshold using the incomplete public library, with the wrong identification rate of ~2% at genus, and 0% at subfamily resolution at the threshold of 90%. It is comparable with a previous study on Sphingidae (Lepidoptera) that 83% of queries could be accurately identified to genus when conspecific barcodes were not represented in the reference library (Wilson et al., 2011). Considering that the number of matched sequences sharply decreased when the identified threshold was raised, a flexible identity threshold should be promoted to maximize biological information for statistical analyses if *nt* databases are adopted for taxonomic assignments in metabarcoding studies, by which short barcodes could be assigned at different taxonomic resolutions.

The ideal reference library is to link each barcode to a voucher specimen, accompanying detailed metadata and reliable taxonomic information (Weigand et al., 2019). Obviously, the current *nt* database failed to meet this criterion according to our result. Though improved taxonomic accuracy of the reference library will significantly enhance barcoding efficiency, this process is tedious and requires the cooperation of international taxonomists, which is becoming rare even among biologists (Curry et al., 2018). Moreover, many erroneous sequences in the public library are impossible to correct due to the lack of voucher specimens. Thus, a local reference library with high taxonomic coverage seems to be a good alternative for barcoding‐based taxonomic assignments in ecological studies. Some studies have shown that better precision and reliability of barcoding results were obtained using comprehensive local databases because they were less prone to introducing taxonomic errors (deWaard et al., 2019). Recently, some regional reference libraries of Chironomidae have been introduced (Gadawski et al., 2022; Kim et al., 2012; Lin, Chang, et al., 2021; Lin, Mo, et al., 2021), but the number is still very limited considering the high species richness of this family.

## 5 SOME SUGGESTIONS FOR FUTURE BARCODING STUDIES OF CHIRONOMIDAE

DNA barcoding has been wildly utilized as an effective tool for species delimitation, life stages association, and biodiversity assessment in Chironomidae studies. Although great achievements have been made during the past two decades, many deficiencies in this field hinder its application for taxonomic assignment in ecological studies. Hebert and Gregory (2005) explained that the standard DNA barcode should meet the requirement of sufficient length, good quality, and comprehensive voucher specimen. In practice, a great number of Chironomidae barcodes deposited in the public database fail to meet this definition due to ambiguous taxonomic information, incomplete metadata, and the absence of digital vouchers. Besides this, the development of barcoding of chironomids has been uneven across different taxa and regions. Some recommendations based on our results are listed here to guide barcoding studies for Chironomidae in the future.

1. Improve the taxonomic coverage and geographic representation of COI barcodes in the public library. With the development of high‐throughput sequencing, it is undoubted that the number of barcodes will soar quickly. However, researchers should be aware that current barcoding efforts are quite unbalanced. Hence, specimens from rare groups and unexplored regions should be prioritized for future barcoding programs to fill the gap in the database.
2. Provide more detailed metadata linked to the uploaded barcodes, especially for digital vouchers and ecological attributes. Sometimes, it is difficult or impossible for the contributors to provide accurate taxonomical identification at high resolution for the submitted specimens. In this case, sharp photos of diagnostic features could be used as important digital vouchers for following taxonomic verification. Surprisingly, ecological attributes are often ignored when contributors submit metadata of barcodes to the databases. However, it is quite meaningful for users to acquire the corresponding ecological data of the matched specimens, such as geographic information and habitat type. Both photos and ecological attributes could be deposited in BOLD, but they have not received enough concern.
3. Call for more rigorous reviews of the taxonomic assignment of barcodes on public databases. The application of barcodes to taxonomic assignment in ecological studies is greatly limited by insufficient and wrong identification in the database. Taxonomists should be motivated to rectify this situation using a two‐pronged approach. On the one hand, they could provide more barcodes with accurate taxonomic information as references for correcting ambiguous identification. On the other hand, they could purge taxonomic errors from databases by checking the digital vouchers and geographical information of the submitted specimens.

## 6 CONCLUSION

1. A COI barcode library of Chironomidae from the TP was curated as an important supplement to the public database. The library comprised 512 barcodes of 159 species from 124 sampling sites, of which many provisional species were likely new to science. The species richness of chironomids is much higher in lotic waterbodies (*n* = 142) than in lentic waterbodies (*n* = 35).
2. The optimal threshold for the molecular identification of Chironomidae from the TP was determined as 2.7%–2.8% K2P genetic distance. The efficiency of barcode‐based identification in the curated library was 96.68% for the family, ranging from 97% to 100% among different subfamilies.
3. The taxonomic coverage of Chironomidae was poorly represented in the public database on the BOLD, including 2836 species and 222 genera. Only a small proportion of public barcodes had been identified at the genus level (52.56%) and species level (29.18%).
4. Barcoding studies of Chironomidae were extremely uneven among different taxa and geographic regions. The subfamilies Chironominae, Orthocladiinae, and Tanypodinae were the most represented taxa, accounting for ~99% of barcodes in the public database. The top 10 countries contributed over 94% of barcodes, while some known biodiversity hotspots lack records.
5. The quality of public barcodes of Chironomidae was of concern. Our results suggested that 19% of species had multiple BINs, 26% had less than three sequences, and 34% shared BINs with other species.
6. The low‐quality public reference library limited the reliability and efficiency of molecular identification. Generally, identification was more efficient and reliable at coarser taxonomic levels, therefor setting flexible identity thresholds is helpful for taxonomic assignment in metabarcoding studies. A reference library of local diversity with robust taxonomic identification is highly recommended for ecosystem assessment and biological monitoring.

## AUTHOR CONTRIBUTIONS

**Wu Han:** Data curation (equal); formal analysis (equal); investigation (equal); methodology (equal); software (equal); writing – original draft (equal). **Hongqu Tang:** Conceptualization (equal); data curation (equal); resources (equal); supervision (equal); validation (equal); writing – review and editing (equal). **Lili Wei:** Data curation (equal); investigation (equal). **Enlou Zhang:** Conceptualization (equal); funding acqusition (lead); resources (equal); supervision (equal); validation (equal); writing – review and editing (equal).

## CONFLICT OF INTEREST STATEMENT

All authors declare that they have no conflict of interest.

## Supporting information


Data S1:

## Data Availability

The list of all specimen records, COI barcodes, trace files, and geographic information is publicly accessible on BOLD (http://v4.boldsystems.org/) through the dataset “DS‐TPCHIR”.
