# Supplementary material for: The first DNA barcode library of Chironomidae from the Tibetan Plateau with an evaluation of the status of the public databases
Source: Ecol Evol. 2023 Feb 27;13(2):e9849. doi: 10.1002/ece3.9849 (PMC9969238; doi:10.1002/ece3.9849)
Supplement: Supplementary file 1 — Data S1: [file ECE3-13-e9849-s001.docx]

**The first DNA barcode library of Chironomidae from the Tibetan Plateau with an evaluation of the status of the public databases**

**Supporting Information**

**Tables**

**Table S1.** Checklist of chironomids species from the TP, with corresponding specimen data. Len. and loc. refer to the lentic and lotic water bodies respectively.

|  | **Subfamily** | **Species** | **Life stage** | **Barcodes (n)** | **BINs(n)** | **Habitats** |
| --- | --- | --- | --- | --- | --- | --- |
| 1 | Chironominae | *Chironomus novosibiricus* | AF | 4 | 1 | Len. |
| 2 | Chironominae | *Chironomus tentans* | AM | 1 | 1 | Len. |
| 3 | Chironominae | *Constempellina* cf. *brevicosta* | AM | 1 | 1 | Len. |
| 4 | Chironominae | *Glyptotendipes barbipes* | AM | 2 | 1 | Len. |
| 5 | Chironominae | *Neozavrelia mongolensis* | AM, P | 2 | 1 | Len. |
| 6 | Chironominae | *Paratanytarsus* sp. T07 | AM | 3 | 1 | Len. |
| 7 | Chironominae | *Paratendipes* sp. T06 | AM | 1 | 1 | Len. |
| 8 | Chironominae | *Paratendipes* sp. TP3 | AM | 1 | 1 | Len. |
| 9 | Chironominae | *Synendotendipes* sp. T10 | L | 2 | 1 | Len. |
| 10 | Chironominae | *Chironomus bernensis* | AM, AF, P, L | 22 | 1 | Len., Lot. |
| 11 | Chironominae | *Chironomus* cf. *striatus* | AM, L | 8 | 1 | Len., Lot. |
| 12 | Chironominae | *Chironomus lugubris* | AM, L | 2 | 1 | Len., Lot. |
| 13 | Chironominae | *Paracladopelma* sp. T04 | AM, P | 2 | 1 | Len., Lot. |
| 14 | Chironominae | *Tanytarsus gracilentus* | AM, P | 4 | 1 | Len., Lot. |
| 15 | Chironominae | *Chironomus* cf. *dorsalis* | L | 1 | 1 | Lot. |
| 16 | Chironominae | *Chironomus* sp. T02 | L | 8 | 1 | Lot. |
| 17 | Chironominae | *Chironomus* sp. TP4 | L | 9 | 3 | Lot. |
| 18 | Chironominae | *Chironomus* sp. TP5 | L | 1 | 1 | Lot. |
| 19 | Chironominae | *Cryptochironomus redekei* | AM, AF | 5 | 1 | Lot. |
| 20 | Chironominae | *Lipiniella* sp. T03 | AM | 1 | 1 | Lot. |
| 21 | Chironominae | *Micropsectra gracelis* | AM, AF, P, L | 10 | 1 | Lot. |
| 22 | Chironominae | *Micropsectra parasofiae* | AM, L | 4 | 2 | Lot. |
| 23 | Chironominae | *Micropsectra* sp. T05 | P, L | 2 | 1 | Lot. |
| 24 | Chironominae | *Micropsectra* sp. TP1 | AM | 1 | 1 | Lot. |
| 25 | Chironominae | *Micropsectra* sp. TP2 | AM | 1 | 1 | Lot. |
| 26 | Chironominae | *Micropsectra* sp. TP42 | P, L | 7 | 1 | Lot. |
| 27 | Chironominae | *Micropsectra* sp. TP43 | L | 1 | 1 | Lot. |
| 28 | Chironominae | *Micropsectra* sp. TP44 | L | 1 | 1 | Lot. |
| 29 | Chironominae | *Micropsectra* sp. TP45 | AM | 1 | 1 | Lot. |
| 30 | Chironominae | *Micropsectra* sp. TP46 | L | 2 | 1 | Lot. |
| 31 | Chironominae | *Micropsectra* sp. TP47 | AM | 1 | 1 | Lot. |
| 32 | Chironominae | *Neozavrelia minuta* | AM | 2 | 1 | Lot. |
| 33 | Chironominae | *Parachironomus* sp. TP8 | AM | 1 | 1 | Lot. |
| 34 | Chironominae | *Paratanytarsus kaszabi* | AM | 2 | 1 | Lot. |
| 35 | Chironominae | *Paratanytarsus setosimanus* | AM, AF, L | 6 | 4 | Lot. |
| 36 | Chironominae | *Polypedilum bullum* | AM, AF, L | 10 | 4 | Lot. |
| 37 | Chironominae | *Polypedilum convexum* | AM | 1 | 1 | Lot. |
| 38 | Chironominae | *Polypedilum dengae* | AF | 1 | 1 | Lot. |
| 39 | Chironominae | *Polypedilum johannseni* | AM | 3 | 2 | Lot. |
| 40 | Chironominae | *Stictochironomus* sp. T08 | P, L | 2 | 2 | Lot. |
| 41 | Chironominae | *Stictochironomus* sp. T09 | AM | 1 | 1 | Lot. |
| 42 | Chironominae | *Tanytarsus sinuatus* | AM | 1 | 1 | Lot. |
| 43 | Chironominae | *Tanytarsus* sp. TP48 | L | 1 | 1 | Lot. |
| 44 | Chironominae | *Tanytarsus* sp. TP49 | AF, L | 4 | 2 | Lot. |
| 45 | Chironominae | *Tanytarsus* sp. TP50 | L | 6 | 1 | Lot. |
| 46 | Diamesinae | *Diamesa* sp. TP12 | AM, L | 2 | 1 | Len., Lot. |
| 47 | Diamesinae | *Pseudodiamesa alica* | AM, AF, P, L | 20 | 3 | Len., Lot. |
| 48 | Diamesinae | *Boreoheptagyia* sp. TP14 | L | 1 | 1 | Lot. |
| 49 | Diamesinae | *Diamesa aberrata* | P | 1 | 1 | Lot. |
| 50 | Diamesinae | *Diamesa amanoi* | L | 2 | 2 | Lot. |
| 51 | Diamesinae | *Diamesa* cf. *aberrata* | L | 5 | 1 | Lot. |
| 52 | Diamesinae | *Diamesa kandzensis* | AM, P, L | 4 | 1 | Lot. |
| 53 | Diamesinae | *Diamesa kaszabi* | AF, L | 9 | 1 | Lot. |
| 54 | Diamesinae | *Diamesa pseudosteinboecki* | AM | 2 | 1 | Lot. |
| 55 | Diamesinae | *Diamesa qiangi* | P, L | 4 | 1 | Lot. |
| 56 | Diamesinae | *Diamesa* sp. T29 | L | 1 | 1 | Lot. |
| 57 | Diamesinae | *Diamesa* sp. T30 | L | 1 | 1 | Lot. |
| 58 | Diamesinae | *Diamesa* sp. T31 | L | 2 | 1 | Lot. |
| 59 | Diamesinae | *Diamesa* sp. TP11 | P | 1 | 1 | Lot. |
| 60 | Diamesinae | *Diamesa* sp. TP52 | L | 2 | 1 | Lot. |
| 61 | Diamesinae | *Pagastia hanseni* | AM, AF, L | 5 | 1 | Lot. |
| 62 | Diamesinae | *Pagastia* sp. T28 | L | 2 | 1 | Lot. |
| 63 | Diamesinae | *Pagastia* sp. TP15 | L | 1 | 1 | Lot. |
| 64 | Diamesinae | *Potthastia* sp. TP16 | P, L | 2 | 1 | Lot. |
| 65 | Diamesinae | *Pseudodiamesa nivosa* | L | 2 | 1 | Lot. |
| 66 | Diamesinae | *Pseudodiamesa* sp. TP13 | AM | 1 | 1 | Lot. |
| 67 | Diamesinae | *Sympotthastia takatensis* | L | 1 | 1 | Lot. |
| 68 | Orthocladiinae | *Acricotopus simplex* | AM | 3 | 1 | Len. |
| 69 | Orthocladiinae | *Cricotopus glacialis* | AM, L | 4 | 2 | Len. |
| 70 | Orthocladiinae | *Limnophyes* sp. T19 | AM | 1 | 1 | Len. |
| 71 | Orthocladiinae | *Orthocladius* sp. TP35 | AF | 1 | 1 | Len. |
| 72 | Orthocladiinae | *Orthocladius* sp. TP36 | AF | 1 | 1 | Len. |
| 73 | Orthocladiinae | *Parakiefferiella* sp. TP22 | AM | 1 | 1 | Len. |
| 74 | Orthocladiinae | *Acricotopus* cf. *lucens* | AM, AF | 10 | 1 | Len., Lot. |
| 75 | Orthocladiinae | *Cricotopus dentatus* | AM, AF, P, L | 16 | 1 | Len., Lot. |
| 76 | Orthocladiinae | *Cricotopus rufiventris* | AM, AF, L | 8 | 1 | Len., Lot. |
| 77 | Orthocladiinae | *Limnophyes bullus* | AM, P, L | 6 | 4 | Len., Lot. |
| 78 | Orthocladiinae | *Orthocladius nitidoscutellatus* | AM, P, L | 15 | 3 | Len., Lot. |
| 79 | Orthocladiinae | *Orthocladius*  *oblidens* | AM, AF, L | 7 | 1 | Len., Lot. |
| 80 | Orthocladiinae | *Paracladius akansextus* | AM, AF, P, L | 15 | 1 | Len., Lot. |
| 81 | Orthocladiinae | *Acricotopus longipalpus* | AM, AF, P, L | 9 | 1 | Lot. |
| 82 | Orthocladiinae | *Acricotopus* sp. T16 | P | 1 | 1 | Lot. |
| 83 | Orthocladiinae | *Acricotopus* sp. T17 | AF | 2 | 1 | Lot. |
| 84 | Orthocladiinae | *Acricotopus* sp. TP38 | L | 1 | 1 | Lot. |
| 85 | Orthocladiinae | *Acricotopus* sp. TP53 | AM, AF, L | 3 | 1 | Lot. |
| 86 | Orthocladiinae | *Acricotopus* sp. TP54 | AM | 1 | 1 | Lot. |
| 87 | Orthocladiinae | *Acricotopus* sp. TP55 | L | 1 | 1 | Lot. |
| 88 | Orthocladiinae | *Acricotopus* sp. TP56 | AM | 1 | 1 | Lot. |
| 89 | Orthocladiinae | *Acricotopus* sp. TP57 | AF | 1 | 1 | Lot. |
| 90 | Orthocladiinae | *Acricotopus* sp. TP58 | L | 5 | 1 | Lot. |
| 91 | Orthocladiinae | *Acricotopus zhalingensis* | AM, AF, P, L | 13 | 3 | Lot. |
| 92 | Orthocladiinae | *Allocladius* sp. T15 | AF | 1 | 1 | Lot. |
| 93 | Orthocladiinae | *Brillia flavifrons* | AM | 1 | 1 | Lot. |
| 94 | Orthocladiinae | *Bryophaenocladius* sp. TP33 | AM | 1 | 1 | Lot. |
| 95 | Orthocladiinae | *Chaetocladius* sp. T22 | AM | 1 | 1 | Lot. |
| 96 | Orthocladiinae | *Chaetocladius* sp. T23 | L | 3 | 1 | Lot. |
| 97 | Orthocladiinae | *Chaetocladius* sp. T24 | P, L | 4 | 4 | Lot. |
| 98 | Orthocladiinae | *Chaetocladius* sp. TP37 | AF | 1 | 1 | Lot. |
| 99 | Orthocladiinae | *Corynoneura arctica* | AM, L | 3 | 1 | Lot. |
| 100 | Orthocladiinae | *Cricotopus* cf. *flavocinctus* | AM, AF, P, L | 6 | 1 | Lot. |
| 101 | Orthocladiinae | *Cricotopus* cf. *skirwithensis* | AM | 1 | 1 | Lot. |
| 102 | Orthocladiinae | *Cricotopus mongolseteus* | AM, L | 3 | 1 | Lot. |
| 103 | Orthocladiinae | *Cricotopus ornatipes* | AM, P, L | 14 | 2 | Lot. |
| 104 | Orthocladiinae | *Cricotopus perniger* | AM, L | 3 | 1 | Lot. |
| 105 | Orthocladiinae | *Cricotopus salinophilus* | P | 1 | 1 | Lot. |
| 106 | Orthocladiinae | *Cricotopus* sp. TP25 | AM, P, L | 3 | 1 | Lot. |
| 107 | Orthocladiinae | *Cricotopus* sp. ZA20 | AM | 1 | 1 | Lot. |
| 108 | Orthocladiinae | *Cricotopus sylvestris* | AM | 1 | 1 | Lot. |
| 109 | Orthocladiinae | *Doithrix* sp. TP31 | AM | 1 | 1 | Lot. |
| 110 | Orthocladiinae | *Eukiefferiella brehmi* | P | 1 | 1 | Lot. |
| 111 | Orthocladiinae | *Eukiefferiella cynae* | AM, P, L | 7 | 1 | Lot. |
| 112 | Orthocladiinae | *Eukiefferiella gracei* | AM | 1 | 1 | Lot. |
| 113 | Orthocladiinae | *Eukiefferiella* sp. TP24 | L | 1 | 1 | Lot. |
| 114 | Orthocladiinae | *Eukiefferiella* sp. TP27 | P | 1 | 1 | Lot. |
| 115 | Orthocladiinae | *Eukiefferiella yasunoi* | P | 1 | 1 | Lot. |
| 116 | Orthocladiinae | *Euryhapsis fuscipropes* | AM | 1 | 1 | Lot. |
| 117 | Orthocladiinae | *Heleniella nebulosa* | AM | 1 | 1 | Lot. |
| 118 | Orthocladiinae | *Heleniella* sp. TP28 | P | 1 | 1 | Lot. |
| 119 | Orthocladiinae | *Limnophyes nudus* | AM, AF, L | 6 | 1 | Lot. |
| 120 | Orthocladiinae | *Metriocnemus* sp. T13 | AF | 1 | 1 | Lot. |
| 121 | Orthocladiinae | *Heleniella curtistila* | AM, AF, P | 3 | 1 | Lot. |
| 122 | Orthocladiinae | *Orthocladius* cf. *oblidens* | L | 1 | 1 | Lot. |
| 123 | Orthocladiinae | *Orthocladius difficilis* | AM, P, L | 6 | 4 | Lot. |
| 124 | Orthocladiinae | *Orthocladius filamentosus* | AM, P, L | 8 | 2 | Lot. |
| 125 | Orthocladiinae | *Orthocladius kanii* | P, L | 3 | 1 | Lot. |
| 126 | Orthocladiinae | *Orthocladius priomixtus* | L | 1 | 1 | Lot. |
| 127 | Orthocladiinae | *Orthocladius* sp. T32 | AF, L | 2 | 1 | Lot. |
| 128 | Orthocladiinae | *Orthocladius* sp. TP19 | L | 1 | 1 | Lot. |
| 129 | Orthocladiinae | *Orthocladius* sp. TP59 | L | 1 | 1 | Lot. |
| 130 | Orthocladiinae | *Orthocladius* sp. TP60 | L | 1 | 1 | Lot. |
| 131 | Orthocladiinae | *Paracladius* sp. T20 | L | 2 | 1 | Lot. |
| 132 | Orthocladiinae | *Parakiefferiella* sp. TP23 | L | 1 | 1 | Lot. |
| 133 | Orthocladiinae | *Parakiefferiella viktana* | AM, L | 2 | 2 | Lot. |
| 134 | Orthocladiinae | *Paraphaenocladius* sp. TP29 | P | 2 | 1 | Lot. |
| 135 | Orthocladiinae | *Psectrocladius barbimanus* | AM, AF | 2 | 1 | Lot. |
| 136 | Orthocladiinae | *Psectrocladius* cf. *sordidellus* | AM | 1 | 1 | Lot. |
| 137 | Orthocladiinae | *Psectrocladius limbatellus* | AM | 1 | 1 | Lot. |
| 138 | Orthocladiinae | *Psectrocladius nevalis* | AM, AF, P, L | 8 | 1 | Lot. |
| 139 | Orthocladiinae | *Psectrocladius* sp. T21 | AF | 1a | 1 | Lot. |
| 140 | Orthocladiinae | *Pseudosmittia* sp. T11 | P, L | 2 | 1 | Lot. |
| 141 | Orthocladiinae | *Rheocricotopus* sp. TP32 | AM, P, L | 4 | 3 | Lot. |
| 142 | Orthocladiinae | *Smittia* cf. *aterrima* | AM | 1 | 1 | Lot. |
| 143 | Orthocladiinae | *Thienemannia* sp. TP30 | L | 1 | 1 | Lot. |
| 144 | Orthocladiinae | *Thienemanniella* sp. T12 | L | 1 | 1 | Lot. |
| 145 | Orthocladiinae | *Thienemanniella triangula* | L | 3 | 2 | Lot. |
| 146 | Orthocladiinae | *Tventenia* cf. *bavarica* | AM, AF, P | 5 | 1 | Lot. |
| 147 | Orthocladiinae | *Tvetenia* sp. TP21 | AM, P | 2 | 1 | Lot. |
| 148 | Podonominae | *Trichotanypus* sp. T27 | AM | 2 | 1 | Lot. |
| 149 | Prodiamesinae | *Odontomesa* sp. T25 | AM | 1 | 1 | Len. |
| 150 | Prodiamesinae | *Monodiamesa bonalpicola* | AM, L | 4 | 1 | Lot. |
| 151 | Prodiamesinae | *Monodiamesa secunditibetica* | AM, AF | 5 | 1 | Lot. |
| 152 | Tanypodinae | *Conchapelopia* sp. TP39 | AF | 1 | 1 | Len. |
| 153 | Tanypodinae | *Procladius choreus* | AM, P | 4 | 1 | Len., Lot. |
| 154 | Tanypodinae | *Radotanypus* sp. T26 | AM, AF | 3 | 1 | Len., Lot. |
| 155 | Tanypodinae | *Ablabesmyia* cf. *phatta* | AM | 1 | 1 | Lot. |
| 156 | Tanypodinae | *Conchapelopia* sp. TP41 | L | 1 | 1 | Lot. |
| 157 | Tanypodinae | *Macropelopia pergrandis* | L | 1 | 1 | Lot. |
| 158 | Tanypodinae | *Procladius choreus* gr. | L | 2 | 2 | Lot. |
| 159 | Tanypodinae | *Tanypus punctipennis* | AM | 1 | 1 | Lot. |

**Table S2.** Summary statistics on the intra-specific and inter-specific genetic distances (K2P) in the curated library

|  | Inter-specific distance (%) | | | Intra-specific distance (%) | | |
| --- | --- | --- | --- | --- | --- | --- |
|  | Min | Max | Average | Min | Max | Average |
| Chironominae | 5.06 | 17.74 | 11.28 | 0.15 | 6.16 | 1.91 |
| Orthocladiinae | 3.84 | 16.74 | 10.26 | 0 | 7.50 | 2.13 |
| Diamesinae | 3.14 | 11.74 | 7.23 | 0.32 | 5.28 | 2.86 |
| Prodiamesinae | 4.08 | 14.60 | 6.34 | 0.77 | 1.24 | 0.96 |
| Tanypodinae | 8.40 | 16.33 | 11.88 | 0.59 | 2.65 | 1.39 |
| Chironomidae | 3.14 | 17.97 | 10.08 | 0 | 7.50 | 2.14 |

**Table S3.** The optimal threshold and barcoding efficiency for molecular identification of chironomids in the newly curated library

|  | Chironomidae | Chironominae | Orthocladiinae | Prodimesinae | Diamesinae | Tanypodinae |
| --- | --- | --- | --- | --- | --- | --- |
| Specimens (ind.) | 512 | 152 | 263 | 10 | 71 | 14 |
| Singletons (ind.) | 74 | 19 | 41 | 1 | 8 | 5 |
| OT (%) | 2.7-2.8 | 4.9-5 | 3.5-3.8 | 0.8-4 | 1.3-2.9 | 2.7-8.3 |
| Cumulative errors | 17 | 0 | 8 | 0 | 2 | 0 |
| No id (ind.) | 91 | 19 | 49 | 1 | 10 | 5 |
| Correct (ind.) | 421 | 133 | 214 | 9 | 61 | 9 |
| Incorrect (ind.) | 0 | 0 | 0 | 0 | 0 | 0 |
| BE (%) | 96.7 | 100 | 97 | 100 | 97.2 | 100 |

Note: Specimens, the number of individuals (ind.) whose barcodes were included in the analyzed dataset; Singletons, the number of singleton species; OT (%), the optimal threshold value for molecular identification determined by threshold optimization analysis; Cumulative errors; the minimum cumulative errors related to OT; No id, the number of sequences fail to match with conspecifics, detected by Best Close Match analysis; Correct, correct identifications; Incorrect, incorrect identifications; BE, the barcoding efficiency estimated for the dataset (singletons were excluded).

**Table S4.** The contribution of the TOP 10 countries/regions to chironomids barcodes on the public database of BOLD

| Country/Region | Specimen (n) | BIN (n) | Genus (n) | Contribution (%) |
| --- | --- | --- | --- | --- |
| Canada | 298,268 | 4,455 | 118 | 60.92 |
| Costa Rica | 82,878 | 1,325 | 25 | 16.93 |
| Australia | 22,543 | 784 | 36 | 4.6 |
| South Africa | 15,167 | 484 | 29 | 3.1 |
| Greenland | 9,811 | 150 | 38 | 2 |
| Norway | 9,042 | 844 | 113 | 1.85 |
| United States | 8,260 | 1031 | 104 | 1.69 |
| Argentina | 5,835 | 445 | 19 | 1.19 |
| Germany | 5,301 | 443 | 70 | 1.08 |
| Honduras | 5,120 | 277 | 4 | 1.05 |

**Table S5.** The number of species included for BAGS analysis and the percentage of species ranked to each grade in each subfamily and family. The letters A to E refer to Grade A to Grade E.

| Group | Species (n) | A (%) | B (%) | C (%) | D (%) | E (%) |
| --- | --- | --- | --- | --- | --- | --- |
| Chironominae | 703 | 7.54 | 11.66 | 19.2 | 25.04 | 36.56 |
| Orthocladiinae | 368 | 8.7 | 6.79 | 20.11 | 30.98 | 33.42 |
| Prodiamesinae | 15 | 6.67 | 33.33 | 20 | 20 | 20 |
| Diamesinae | 60 | 6.67 | 21.67 | 10 | 30 | 31.67 |
| Telmatogetoninae | 4 | 0 | 0 | 25 | 75 | 0 |
| Tanypodinae | 146 | 14.38 | 19.86 | 15.75 | 18.49 | 31.51 |
| Podonominae | 12 | 0 | 16.67 | 16.67 | 41.67 | 25 |
| Chironomidae | 1308 | 8.49 | 11.92 | 18.65 | 26.45 | 34.48 |

**Figure and legend**


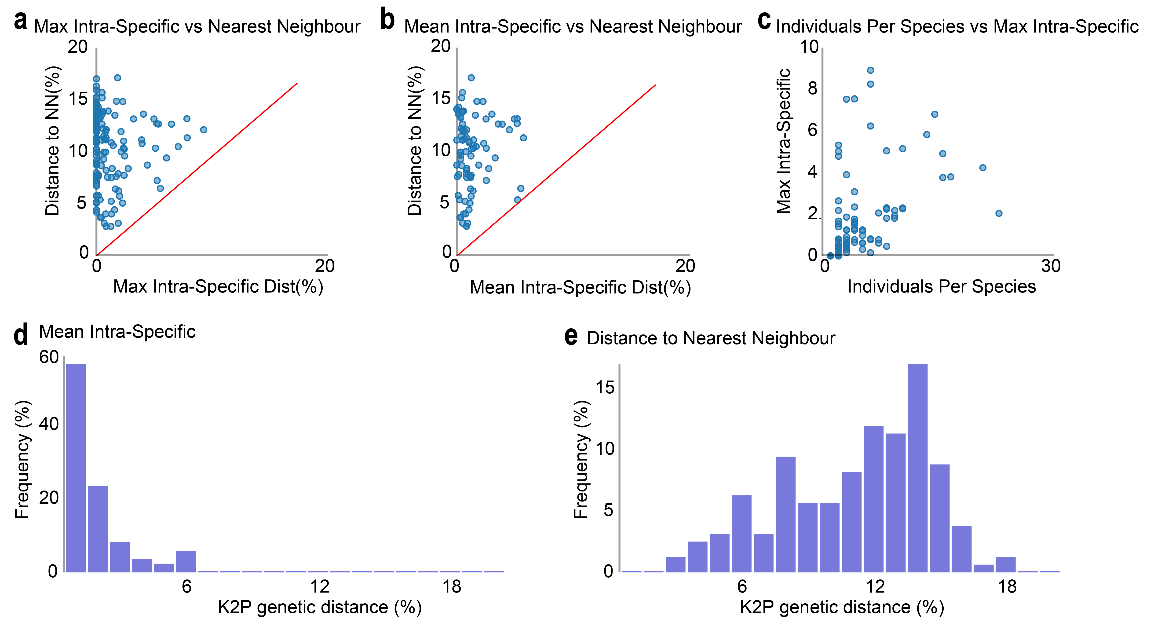


**Figure S1.** Barcode Gap Analysis on the 512 COI barcodes of Chironomidae from the TP. (a-b), the first two scatterplots show the overlap of the max and mean intra-specific distance vs. inter-specific (nearest neighbor) distances; (c) the third scatterplot shows the number of individuals in each species against their max intra-specific distances as a test for sampling bias. The two histograms show the distribution of mean intra-specific distances (d) and inter-specific distances to the nearest neighbor (e).
